# Mouth Breathing and Its Impact on Atypical Swallowing: A Systematic Review and Meta-Analysis

**DOI:** 10.3390/dj12020021

**Published:** 2024-01-23

**Authors:** Carmen Gómez-González, Antonio González-Mosquera, Mohammad Hamdan Alkhraisat, Eduardo Anitua

**Affiliations:** 1 Clínica Antonio González Mosquera, Avenida Linares Rivas, 26, 15005 A Coruña, Spain; 2 Regenerative Medicine Department, BTI Biotechnology Institute, Jacinto Quincoces 39, 01007 Vitoria-Gasteiz, Spain; mohammad.hamdan@bti-implant.es (M.H.A.); eduardo@fundacioneduardoanitua.org (E.A.); 3 University Institute for Regenerative Medicine & Oral Implantology, UIRMI (UPV/EHU-Fundación Eduardo Anitua), 01007 Vitoria-Gasteiz, Spain

**Keywords:** mouth breathing, oral breathing, tongue thrust, atypical swallowing, tongue habits

## Abstract

The aim of this systematic review is the assessment of the effect of mouth breathing on the prevalence of tongue thrust. The review was performed according to the PRISMA 2020 checklist guidelines, and the protocol was registered with PROSPERO (CRD42022339527). The inclusion criteria were the following: studies of clinical trials and cross-sectional and longitudinal descriptive studies that evaluate the appearance of tongue thrust in patients with mouth breathing; healthy subjects of any age, race or sex; and studies with a minimum sample group of five cases. The exclusion criteria were the following: studies with syndromic patients, articles from case reports, and letters to the editor and/or publisher. Searches were performed in electronic databases such as The National Library of Medicine (MEDLINE via PUBMED), the Cochrane Central Register of Controlled Trials, Web of Science and Scopus, including studies published until November 2023, without a language filter. The methodological quality of the included case–control studies was assessed using the Newcastle–Ottawa Scale (NOS), and the Joanna Briggs Institute (JBI) tool was used for descriptive cross-sectional studies and cross-sectional prevalence studies. A meta-analysis was conducted on studies that provided data on patients’ classification according to mouth breathing (yes/no) as well as atypical swallowing (yes/no) using Review Manager 5.4. From 424 records, 12 articles were selected, and 4 were eligible for meta-analysis. It was shown that there is no consensus on the diagnostic methods used for mouth breathing and tongue thrust. The pooled risk ratio of atypical swallowing was significantly higher in the patients with mouth breathing (RR: 3.70; 95% CI: 2.06 to 6.66). These studies have several limitations, such as the heterogeneity among the individual studies in relation to the diagnostic tools and criteria for the assessment of mouth breathing and atypical swallowing. Considering the results, this systematic review shows that patients with mouth breathing presented higher risk ratios for atypical swallowing.

## 1. Introduction

Patients with mouth breathing are at risk of developing altered dental and facial skeletal growth [1,2], sleep disorders [3] and poor quality of life [4,5,6]. The etiological factors of this common condition could be divided into obstructive and functional factors [7,8]. Tonsillar hypertrophy, deviated nasal septum and the presence of nasal polyps are among the obstructive factors. Meanwhile, functional factors include prolonged oral habits, muscular alterations or transient edema of the nasal mucosa (allergic rhinitis) [7,8].

Nasal breathing is a key factor in the correct development of the oral cavity [9,10]. When mouth breathing occurs, the lips remain open, the contraction of the mandibular elevator muscles is reduced, the perioral muscles’ activity is triggered and a lower or anterior position of the tongue is adopted. The consequences of these changes could be the development of a long face, narrow maxilla, high-arched palate, class II or class III skeletal profiles, anterior open bite, anterior or posterior crossbite, short upper lip, everted lower lip and forward head posture, among others [1,7,10,11,12,13,14].

Mouth breathing has also been associated with tongue thrust or atypical swallowing [15,16,17]. Tongue thrust consists of the introduction and support of the tongue between the incisors during swallowing. Tongue thrust has been described as a risk factor for the appearance of malocclusions associated with the proclination of the upper anterior teeth, anterior open bite, crossbite and high and/or narrow palate [1,18,19]. The tongue plays a very important role in many oral functions, such as swallowing, chewing, phonation and breathing [15]. The appearance of alterations in the tongue can alter these functions.

For all these reasons, the importance of adequate management and early diagnosis of patients with mouth breathing and tongue thrust habits is evident in order to avoid or minimize their impact on the development of the dental–facial complex. However, there is a need for an assessment of the clinical diagnostic tools used to establish these two conditions and for an estimate of the association of mouth breathing with tongue thrust or atypical swallowing. Thus, a systematic review is needed to achieve these objectives and critically appraise the available studies.

The aim of this systematic review is to analyze the impact of the persistence of mouth breathing on the appearance of the tongue thrust habit in terms of prevalence, assessing the diagnostic tools and assessing the quality of the available evidence.

## 2. Materials and Methods

### 2.1. Protocol

The protocol of this systematic review was listed in a public registry (the PROSPERO database) under reference number CRD42022339527. The PRISMA guidelines were followed in the reporting of this manuscript [20].

The main question of this systematic review was as follows: what is the prevalence of the tongue thrust habit (comparison and outcome) in the general population (population) with a diagnosis of mouth breathing (exposition)?

The search strategy was defined by the Population, Intervention, Comparison and Outcomes (PICO) question:

- P = General population without syndromes;
- I = Patients with a diagnosis of mouth breathing;
- C = Presence or not of tongue thrust;
- O = Prevalence of tongue thrust or atypical swallowing.

Electronic searches were performed in The National Library of Medicine (MEDLINE via PUBMED), Cochrane Central Register of Controlled Trials, Web of Science and Scopus. Studies published until May 2022 were included. The search was not limited by any language filter.

The following search terms were used:

- MEDLINE and CENTRAL:
  - Exposition: (mouth breathing [Mesh] OR mouth breathing [Title/Abstract] OR oral breathing [Title/Abstract]).
  - Comparation: (tongue habits [Title/Abstract] OR tongue habits [Mesh] OR atypical swallowing [Title/Abstract] OR tongue thrust [Title/Abstract]).
- WOS and SCOPUS:
  - Exposition: (mouth breathing OR oral breathing).
  - Comparation (tongue habits OR atypical swallowing OR tongue thrust).

The references contained in all publications included were cross-checked to identify any relevant publications missing from the search.

### 2.2. Selection of Studies

All titles and abstracts obtained from the search were screened independently by two reviewers (C.G.-G. and A.G.-M.) according to pre-set eligibility criteria. Papers with insufficient data in the title and abstract were selected for exclusion. Full texts of these publications were evaluated to determine the final decision for inclusion/exclusion. Any disagreement was resolved through discussion with a third reviewer (M.H.A.). Reasons for rejecting studies based on the full-text evaluation were recorded in a data table.

The reliability of the extracted data between reviewers was determined using the Kappa index.

The inclusion criteria were the following:

- Studies of clinical trials and cross-sectional and longitudinal descriptive studies that evaluate the appearance of tongue thrust in patients with mouth breathing;
- Healthy subjects of any age, race or sex;
- Studies with a minimum sample group of 5 cases.
- The exclusion criteria were the following:
- Studies with syndromic patients;
- Articles from case reports, letters to the editor and/or publisher.

In the case of multiple publications conducted on the same study population, only the study with the longest follow-up time was included.

### 2.3. Data Extraction

The full texts of the preliminarily selected studies were obtained and evaluated in order to verify that they met all the inclusion criteria. In specific cases, the authors of the potentially eligible articles were contacted by email and information on the eligibility of the article was requested.

Data were extracted independently by the two reviewers (C.G.-G. and A.G.-M.) using custom data extraction tables. Any disagreements were resolved via discussion with a third review author (M.H.A.).

Reasons for rejecting studies at this stage or at later stages were recorded.

### 2.4. Risk of Bias and Methodological Quality of the Studies

The two review authors (C.G.-G. and A.G.-M.) independently assessed the risk of bias in the included studies. Disagreements about the risk of bias in particular studies were resolved via discussion between the two review authors, and a third reviewer was consulted when necessary (M.H.A.).

The methodological quality of the included case–control studies was assessed using the Newcastle–Ottawa Scale (NOS) [21]. The Joanna Briggs Institute (JBI) tool was used for descriptive cross-sectional studies and cross-sectional prevalence studies [21].

The results are provided in Appendix A: Table A1, Table A2 and Table A3.

### 2.5. Data Register

The following data were extracted from full-text publications: author(s); year of publication; type of study; population (adults or children); age, sex and race; sample size, including the number of patients included and number of patient dropouts; follow-up time; signs and symptoms of mouth breathing; mouth breathing assessment method (i.e., measured by clinical assessment and validated tests); signs and symptoms of tongue thrust; tongue thrust assessment method (i.e., measured by clinical assessment and validated tests); other associated anatomical parameters such as anterior open bite, posterior crossbite, bruxism/parafunction, phonetics and apnea; and other habits such as finger sucking, lip sucking and bottle feeding. Authors of the selected studies were contacted to disclose their data in a 2 × 2 table according to mouth breathing (yes/no) and atypical swallowing (yes/no).

### 2.6. Statistical Analysis

The studies that provide data on patients’ classification according to mouth breathing (yes/no) as well as atypical swallowing (yes/no) were included in a meta-analysis, performed using Review Manager 5.4 (The Nordic Cochrane Centre, Copenhagen, Denmark). A pooled risk ratio (RR; 95% confidence interval) was calculated using the Mantel–Haenszel method. The similarity of the estimated RR and the 95% CI of the individual studies were assessed. The I2 statistic was calculated to assess the heterogeneity of the included studies. An I2 value higher than 75% pointed out the presence of substantial heterogeneity. The absence of statistically significant heterogeneity indicated the use of a fixed-effects model. Otherwise, a random-effects model was used. Forest plots were created to represent the meta-analysis outcomes. Funnel plot analysis could not be performed due to the limited number of studies.

### 2.7. Updated Searches

A search update was performed in November 2023. The papers identified (*n* = 5) were subjected to the same scrutiny as for the initial search.

## 3. Results

### 3.1. Literature Search and Quality of the Papers

**Figure 1:**
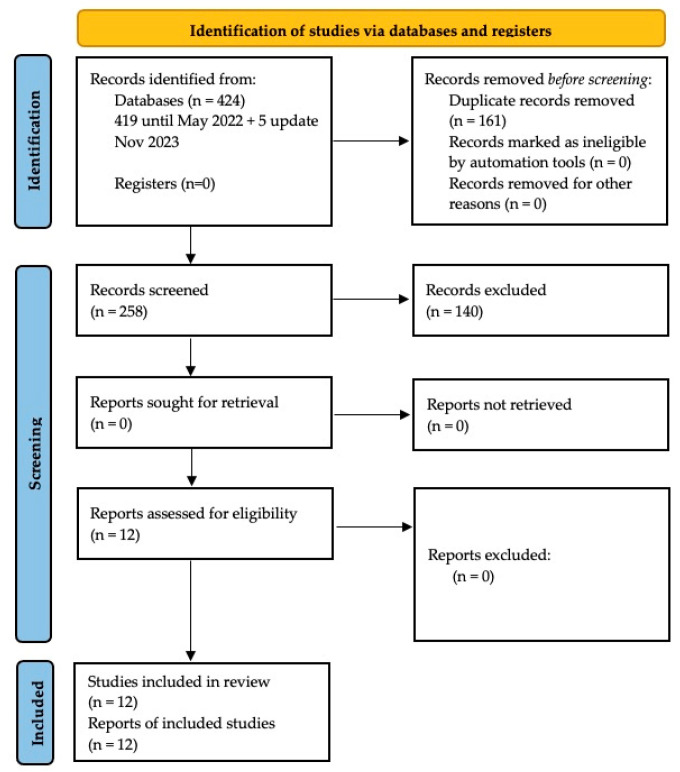
Flow chart diagram of study selection process.

In the initial search, 419 articles were obtained, of which 3 were found in Cochrane, 127 in Pubmed, 138 in Scopus and 151 in WOS. Of these, 140 were duplicates (Figure 1). After observing all the inclusion criteria, 12 articles were selected for qualitative synthesis. An email with a 2 × 2 table was sent to the authors of the included articles that presented incomplete data or data that did not relate the subjects with mouth breathing and tongue thrust to each other. See Figure 1 for the PRISMA flowchart.

Of the 12 articles, 2 were case–control studies and were evaluated using the Newcastle–Ottawa Scale (NOS). Both were given a score of 7 [15] and 5 [22] out of 8 items (Appendix A: Table A1). The other 10 were descriptive cross-sectional studies and cross-sectional prevalence studies, and they were eligible for critical appraisal using the JBI appraisal tools (Appendix A: Table A2 and Table A3). The authors scored each item as “yes”, “no”, “unclear”, or “not applicable” when assessing the quality of each included study. Decisions about scoring were discussed by two reviewers (C.G.-G. and A.G.-M.), and a third reviewer was consulted when necessary (M.H.A.). In order to avoid oversight, it was considered apposite to include all 12 papers in the review, although 5 of them would require the reviewers to seek out more information [16,23,24,25,26].

Finally, 12 articles were included, 4 of which classified patients according to mouth breathing and atypical swallowing.

### 3.2. Characteristics of the Selected Studies

Table 1 summarizes the characteristics of the included studies. Regarding the year of publication, the highest concentration of studies appeared between the years 2012 and 2015 [16,19,22,27,28], mainly in the year 2013 [16,27,28]. Two of the studies were conducted in India [15,28], two in Italy [17,29] and the rest were carried out in different countries: Brazil [26], Argentina [16], Albania [27], Lithuania [19], Romania [22], Ecuador [25], Pakistan [23] and Peru [24]. Regarding the type of study, most were cross-sectional observational and two were case–control studies, one of them cross-sectional [15] and another retrospective [22]. The ages of the sample ranged from 3 to 20 years, and only one study specified the races of the sample participants [27]. The qualitative characteristics of the studies are presented in Table 1.

Regarding the tests carried out for the diagnosis of mouth breathing, a variety of methods were used. The most-used method was direct clinical observation [15,16,17,19,23,24,25,27,28,29]; however, only two of the studies describe in detail how such direct observation was performed [17,24]. Another method used was the completion of a questionnaire, most of which were administered to parents [19,26,29], although one was administered to the children [27]. In some cases, both observational and questionnaire methods were combined [19,27,29]. Furthermore, in the studies of Shetty et al. [28] and González et al. [25], a mirror was used to diagnose the mouth breathing of the subjects. The characteristics of the methods used in each study for the diagnosis of oral respiration are described in Table 2.

Regarding the tests carried out for the diagnosis of tongue thrusting, a variety of methods were also observed. The most used method was direct clinical observation. Several studies diagnosed the presence of tongue thrust if subjects presented contraction of the perioral musculature when swallowing [15,17,19,24,26]; however, these studies used different criteria for their evaluation. Melsen et al. [17] observed the mandibular movement in patients and palpation of the masseter and temporal muscles when swallowing saliva or small sips of water. Three studies [19,24,26] considered that atypical swallowing occurred when, in addition to contraction of the perioral musculature, the tip of the tongue was placed between the anterior teeth when swallowing saliva three times [19] or if the patient spilled water when drinking it from a glass [24]. Two of the studies [15,28] diagnosed the presence of tongue thrusting if the subjects met any of the criteria established by Weiss and Van Houten [30] when swallowing 10 mL of water. Two studies used a disclosing solution to observe the trace left by the tongue when swallowing [15,25]. Three studies were also aided by questionnaires, two of them administered to parents [19,29] and one to the children [27]. Knösel et al. [16] first observed the habit of swallowing saliva with open lips and later performed a polysensography test. Zegan et al. did not report their diagnostic assessment method [22]. The characteristics of the methods used in each study for the diagnosis of thrusting of the tongue are described in Table 3.

Among the studies that met the inclusion and exclusion criteria of the review, it was observed that four articles presented data that related mouth breathing to tongue thrust. Moreover, Rodríguez-Olivos et al. provided us their data within the 2 × 2 table that had been sent to the corresponding author [24]. Melsen et al. studied the relationship between the swallowing pattern, the mode of breathing and the appearance of malocclusions. From an initial sample of 824 children, 40 presented mouth breathing, of which 77.5% (*n* = 31) presented the habit of tongue thrusting [17]. Knosel et al. selected a sample of 29 children who had the habit of having their mouths open during the day and, in addition, complied with the typical facial characteristics of the mouth respirator. They observed that the vast majority (*n* = 27) presented atypical swallowing [16]. Dixit et al. conducted a case–control study with the objective of analyzing and comparing the morphological characteristics of soft, dental and skeletal tissues in children with and without the habit of tongue thrusting. In the control group, no child presented mouth breathing; however, in the group of children with tongue thrust, 38% presented mouth breathing [15]. The objective of the study by Noor et al. was to analyze the possible relationship of different modes of breathing (oral, nasal and combined) with different malocclusions. From a total sample of 62 subjects, 24 patients had mouth breathing, 18 had nasal breathing and 20 had combined or mixed breathing. Moreover, 33.3% (*n* = 8) of mouth breathers presented atypical swallowing [23]. Rodríguez-Olivos et al. evaluated the relationship of dental malocclusions with different habits acquired in children between 6 and 12 years of age. They provided us with data on the subjects’ mode of breathing and swallowing. From a total of 156 children evaluated, 10 presented oral breathing, of which more than half (*n* = 6) presented atypical swallowing [24]. These data are detailed in Table 4.

**Figure 2:**
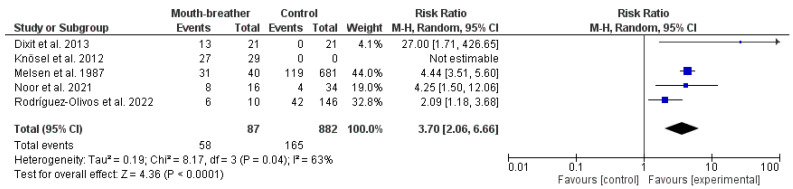
Meta-analysis of the studies providing data on mouth breathing and atypical swallowing [15,16,17,23,24]. (CI: confidence interval; I^2^: heterogeneity index).

Figure 2 shows a forest plot representing the results of the meta-analysis. The pooled risk ratio of atypical swallowing was significantly higher in patients with mouth breathing (RR: 3.70; 95% CI: 2.06 to 6.66).

## 4. Discussion

Chronic mouth breathing could predispose patients to several functional and morphological adaptations that would affect their posture, auditory processing and lung performance [31,32,33,34]. Additionally, the space of the upper airway is shortened by mouth breathing due to the reductions in the mandible–hyoid bone distance, retropalatal area and retroglossal area [35]. The narrowing in the upper airway space could provoke an obstructive sleep apnea. Kuroishi et al. have shown that mouth-breathing children had lower cognitive performance in reading comprehension, arithmetic and working memory for pseudowords [32]. Nasal breathing is a route that could modular the cognitive function as it connected to the limbic areas of the brain that mediate emotion, memory and behavior [36]. It improves the reaction time toward a threat and the recognition of visual objects [36].

In this systematic review, different diagnostic methods used for mouth breathing are observed. That is, there is no unified evaluation method to detect these habits. Often, patients with mouth breathing are associated with typical features of “adenoid facies”, such as labial incompetence, an open-mouthed posture, a high-arched palate, a narrow jaw and a long face, among others. In addition, Fraga et al. confirmed in their systematic review that mouth breathing is also related to dental malocclusions [37]. Most of the selected studies made direct clinical observation of the factors associated with mouth breathing [15,16,17,19,23,24,25,27,28,29], for example, in the study by Melsen et al., they checked if there was a lip seal to identify the subjects with mouth breathing [17]. However, the relationship between an incompetent lip seal and mouth breathing is unclear. In a similar study, Ovsenik [38], in addition to observing if there was a lip seal, verified the mode of breathing with an airflow recording device to correctly differentiate mouth breathing from an incompetent lip seal habit. Other authors [19,27,29] used questionnaires to diagnose respiration. Castelo et al. administered a questionnaire to parents and measured the presence of qualitative (yes/no) and quantitative (frequent/occasional/never) oral breathing [26]. Other studies, such as that of Laganà et al., provided the questionnaire directly to the children [27]. Another diagnostic method found was a mirror test [25,28], which normally assesses the degree of cloudiness in a mirror placed under the nose. In order to present a unified diagnostic method of oral respiration without the use of special devices, Sano et al. developed a questionnaire as well as a list of items to consider for the visual assessment [39].

Atypical swallowing is related to an altered position of the tongue during swallowing [40]. It is characterized by its high incidence and the multifactorial etiology. It has been linked to malocclusion as a causative or exacerbating factor, emphasizing the need for early diagnosis and treatment through a multidisciplinary approach by combining orthodontic treatment and myofunctional rehabilitation [40].

With respect to the diagnostic methods used in tongue thrusting, the same thing occurs; that is, there is no consensus and the content of the evaluation employed to detect it is left to the discretion of the dentist. Subjects with a habit of tongue thrusting or atypical swallowing, in addition to interposing the tongue between the incisors when swallowing, show a different activation of the perioral muscles with respect to subjects with a normal/adult swallowing pattern [41]. Several of the selected studies observed whether subjects presented contraction of the perioral musculature when swallowing [15,17,19,24,26]; however, they used different criteria for their evaluation. Two of the studies [15,28] diagnosed the presence of tongue thrusting if the subjects met any of the criteria established by Weiss and Van Houten [30]. Dixit et al. and González et al. used a revealing solution to observe the trace left by the tongue when swallowing [15]. Knösel et al. first evaluated the existence of tongue thrusting by asking the subject to swallow saliva with open lips and then performed a polysensography test [16]. They concluded that assessing tongue thrusting habit from direct observation of open lips is questionable. More recent studies, such as that of Kurihara et al., use direct observation of the lingual interposition between the anterior teeth for the diagnosis of atypical swallowing, as well as study the strength of the tongue from electropalatography devices [42].

Regarding the influence of mouth breathing on the occurrence of tongue thrust, Melsen et al. observed that 77.5% of mouth breathing patients had the habit of tongue thrust [17]. Rodríguez-Olivos et al. determined that out of a total of 156 children, 10 presented mouth breathing, of which more than half (*n* = 6) presented atypical swallowing [24]. These results differ from those found in the Noor study, where 33.3% of mouth breathers presented atypical swallowing [23]. These differences in results between studies may be due to heterogeneity regarding diagnostic methods and criteria for the evaluation of oral breathing and atypical swallowing.

Mouth breathing has been included in the list of respiratory problems that would precipitate atypical swallowing, alongside tonsillar and adenoid hypertrophy [43]. Swallowing and respiration are two highly coordinated functions which prevent pulmonary inspiration [44]. Due to the lack of lip seal, mouth breathing is often accompanied by anterior lingual interposition or tongue thrust to produce the seal required to start swallowing [16]. The results of this systematic review support the close relationship between lingual interposition and mouth breathing in light of the high incidence of atypical swallowing in mouth-breathing patients.

These studies have several limitations that include the heterogeneity among the individual studies in relation to the diagnostic tools and criteria for the assessment of oral breathing and atypical swallowing. Differences in race and their influence on the outcomes could not be assessed. There has been a limited number of studies that disclose the outcomes according to the type of breathing and the type of swallowing.

## 5. Conclusions

Patients with oral breathing presented a higher risk ratio for atypical swallowing. Standardization of diagnostic tools and criteria for the assessment of the two conditions would enhance the reliability of the assessment of the association between oral breathing and atypical swallowing.

## Figures and Tables

**Table 1:** Main characteristics of the studies that meet the inclusion criteria for qualitative analysis.

Author, Year, Location, Language of Publication	Place	Sample	Male	Female	Age	Race
Melsen et al. (1987) [17], Italy (English).	Trento village school (Italy)	824 children	424 male	400 female	13–14 years	Not specified
Castelo et al. (2005) [26], Brasil (English).	Piracicaba (Brasil)	99 children	58 male	41 female	3–5 years	Not specified
Knösel et al. (2012) [16], Argentina (English).	Two orthodontic centers in Santa Fé (Argentina)	29 children	16 male	13 female	6–16 years	Not specified
Dixit et al. (2013) [15], India (English).	City of Bagalkot (India)	- Initial sample: 864 children - Study sample: 42 children	27 male - Control group: 21 children: 17 male - Tongue thrust group: 21 children: 10 male	15 female - Control group: 21 children: 4 female - Tongue thrust group: 21 children: 11 female	8–14 years	Not specified
Laganà et al. (2013) [27], Albania (English).	15 public schools in Tirana (Albania)	2617 children	1257 male (48.4%)	1360 female (51.6%)	7–15 years	Exclusion criteria: non-Albanian people
Shetty et al. (2013) [28], India (English).	Department of Pediatrics in Rajnandgaon, (India)	1891 children	1043 male	848 female	6–11 years	Not specified
Kasparaviciene et al. (2014) [19], Lithuania (English).	17 day care centers (Lithuania)	503 children	260 male	243 female	5–7 years	Not specified
Zegan et al. (2015) [22], Romania (English).	Orthodontic Clinic of “St. Spiridon” University Emergency Hospital Iasi (Romania)	525 children	217 male	308 female	6–18 years	Not specified
Caruso et al. (2019) [29], Italy (English).	University of l’Aquila (Italia)	198 children	96 male	102 female	3–5 years	Not specified
González et al. (2020) [25], Ecuador (Spanish).	Cuenca city school, (Ecuador)	53 children	22 male	31 female	5–12 years	Not specified
Noor et al. (2021) [23], Pakistan (English).	Department of Orthodontics, Ayub Medical College, Abbottabad, (Pakistan)	62 children and adults	29 male	33 female	6–20 years	Not specified
Rodríguez-Olivos et al. (2022) [24], Peru (English).	Undergraduate Clinic of the Faculty of Dentistry of the National University of San Marcos, (Peru)	155 children	75 male	80 female	6–12 years	Not specified

**Table 2:** Diagnostic methods used in the studies for the evaluation of mouth breathing.

Author, Year	*n*	Mouth Breathing Evaluation Method	Results
Melsen et al. (1987) [17]	824 children	Observational: Two operators observed whether the patient had a lip seal in a relaxed position. If this was not the case, the child was asked to close their lips and breathe deeply through their nose. If there was a contraction in the perioral muscles or the patient had difficulty breathing, they were asked where they usually breathed, through the mouth or through the nose. The breathing pattern was only collected if the patient’s version coincided with what was observed by the operators.	40 presented mouth breathing
Castelo et al. (2005) [26]	99 children	Questionnaire for parents: presence of qualitative (yes/no) and quantitative (frequent/occasional/never) mouth breathing.	37 presented mouth breathing
Knösel et al. (2012) [16]	29 children (who had an open mouth habit during the day)	Direct clinical observation (not specified).	29 presented mouth breathing
Dixit et al. (2013) [15]	- Initial sample: 864 children - Study sample: 42 children	Direct clinical observation (not specified).	Of the 21 children with the tongue thrusting habit, 38% presented mouth breathing
Laganà et al. (2013) [27]	2617 children	Direct clinical observation (not specified) + questionnaire administered to children.	613 presented mouth breathing (303 male, 310 female)
Shetty et al. (2013) [28]	1891 children	A calibrated examiner. Tried using a mirror.	246 presented mouth breathing
Kasparaviciene et al. (2014) [19]	503 children	Questionnaire for parents + extraoral examination of the face (a single examiner). The mouth breathing diagnostic test was only performed when the general clinical examination indicated mouth breathing and the parents confirmed the presence of the habit in the questionnaires.	51 presented mouth breathing (32 male, 19 female)
Zegan et al. (2015) [22]	525 children	Not described.	34 presented mouth breathing
Castelo et al. (2019) [29]	198 children	Questionnaire for parents + clinical examination by an orthodontist with more than 5 years of experience, calibrated. They used a protocol that they do not describe.	71 presented mouth breathing
González et al. (2020) [25]	53 children	Interview + facial and dental examination + Glatzel mirror.	18 presented mouth breathing
Noor et al. (2021) [23]	62 children and adults; 29 male 33 female	Clinical examination and medical history. Not specified.	Of the total sample: 51.50% of the women and 24.10% of the men presented mouth breathing; mixed breathing (mouth and nasal) 15.20% of women and 51.70% of men
Rodríguez-Olivos et al. (2022) [24]	155 children	Observational: nasal breathing: tape was attached to the nasal septum that had two cotton pads, one in each nostril, and the movement was observed. Mouth breathing: observed napkin movement in a cut mask.	10 presented mouth breathing

**Table 3:** Diagnostic methods used in the studies for the evaluation of tongue thrusting.

Author, Year	*n*	Tongue Thrust Evaluation Method	Results
Melsen et al. (1987) [17]	824 children	Observational: Two operators observed mandibular movement and perioral muscle contraction when swallowing saliva or small sips of water. They then palpated the temporalis and masseter muscles while the patient repeated the process. If they had any doubt, the test was repeated.	60 children presented simple tongue thrust and 90 complex tongue thrust. A total of 150 presented lingual interposition.
Castelo et al. (2005) [26]	99 children	Observational: Two operators. Atypical swallowing was considered to occur when the activity of the lips produced strong tension in the perioral musculature and/or the tip of the tongue placed or pushed against the anterior teeth during swallowing.	29 presented tongue thrust.
Knösel et al. (2012) [16]	29 children (who had an open mouth habit during the day)	1. Observational: patient swallowed saliva with open lips. 2. Polysensography: intraoral sensors in individualized splints were placed on the palate to perform simultaneous measurements of optical distance between the tongue and the palate.	27 presented tongue thrust.
Dixit et al. (2013) [15]	- Initial sample: 864 children - Study sample: 42 children	For 864 patients in the initial sample: The child was asked to first swallow saliva and then 10 mL of water. The position of the tongue during swallowing was assessed by pressing the infant’s lower lip with the operator’s thumbs and at the same time feeling the activity of the masseter muscle with the index fingers. The child was diagnosed with tongue protrusion if they met any of the criteria established by Weiss and Van Houten [30]. For the tongue thrust group (21): The position of the tip of the tongue during swallowing was determined by covering the tip of the tongue with a developer solution with a brush and asking the child to swallow their own saliva. The area of the palate or teeth that was stained was noted. The presence or absence of clefts in the tongue was also recorded.	46 presented tongue thrust.
Laganà et al. (2013) [27]	2617 children	Direct clinical observation (not specified) + questionnaire administered to children.	424 presented tongue thrust (189 male, 235 female).
Shetty et al. (2013) [28]	1891 children	A calibrated examiner. The child was asked to first swallow saliva, and then 10 mL of water. The position of the tongue during swallowing was assessed by pressing the infant’s lower lip with the operator’s thumbs and at the same time feeling the activity of the masseter muscle with the index fingers. The child was diagnosed with tongue thrust if he met any of the following criteria established by Weiss and Van Houten [30].	329 presented tongue thrust.
Kasparaviciene et al. (2014) [19]	503 children	Questionnaire for parents + extraoral examination of the face (a single examiner). The presence of tongue thrust was considered when there was hyperactivity of the perioral muscles and protrusion of the tongue between the upper and lower incisors or canines, without molar contact. Children were asked to swallow 3 times during the same visit. When in doubt, another drink was requested until the observer was satisfied with the judgement.	27 presented tongue thrust (7 male, 20 female).
Zegan et al. (2015) [22]	525 children	Not described.	10 presented tongue thrust.
Caruso et al. (2019) [29]	198 children	Questionnaire for parents + clinical examination by an orthodontist with more than 5 years of experience, calibrated + protocol that is not described.	32 presented tongue thrust.
González et al. (2020) [25]	53 children	Interview + facial examination + Payne’s test.	19 presented tongue thrust.
Noor et al. (2021) [23]	62 children and adults; 29 male, 33 female	Clinical examination and medical history. Not specified.	12 presented tongue thrust.
Rodríguez-Olivos et al. (2022) [24]	155 children	Glass of water + observe muscle contraction + see if water comes out of the mouth or if tongue is in interposition when swallowing. The swallowing process was also observed using oral retractors and introducing a little water with an injector.	51 presented tongue thrust.

**Table 4:** Patients with oral breathing presenting atypical swallowing, 2 × 2 table.

	Tongue Thrust										
		Dixit et al. [15]		Melsen et al. [17]		Knosel et al. [16]		Noor et al. [23]		Rodríguez-Olivos et al. [24]	
Mouth breathing		Yes	No	Yes	No	Yes	No	Yes	No	Yes	No
	Yes	13	8	31	9	27	2	8	16	6	4
	No	0	21	119	562	-	-	4	34	45	101

## Data Availability

Not applicable.

## Appendix A

**Table A1:** The Newcastle–Ottawa Scale (NOS) for case–control studies. To be included, a study can be awarded a maximum of one star for each numbered item within the Selection and Exposure categories; a maximum of two stars can be given for Comparability.

The Newcastle–Ottawa Scale (NOS) for Case–Control Study			
Author		Dixit et al. 2013 [15]	Zegan et al. 2015 [22]
Selection: (Maximum 4 stars)	1. Is the case definition adequate?	☆	☆
	2. Representativeness of the cases	☆	☆
	3. Selection of Controls	☆	/
	4. Definition of Controls	☆	☆
Comparability: (Maximum 2 stars)	5. Comparability of cases and controls on the basis of the design or analysis	☆	☆
Outcome: (Maximum 3 stars)	6. Ascertainment of exposure	☆	/
	7. Same method of ascertainment for cases and controls	☆	☆
	8. Non-response rate	/	/
Total score =		7	5

**Table A2:** The Joanna Briggs Institute (JBI) Critical Appraisal Checklist for analytical cross-sectional studies.

The Joanna Briggs Institute (JBI) Critical Appraisal Checklist for Analytical Cross-Sectional Studies					
Authors	Castelo et al. 2005 [26]	Knosel et al. 2012 [16]	González et al. 2020 [25]	Noor et al. 2021 [23]	Rodriguez-Olivos et al. 2022 [24]
1. Were the criteria for inclusion in the sample clearly defined?	Yes	Yes	No	Yes	Yes
2. Were the study subjects and the setting described in detail?	Yes	Yes	Yes	Yes	Yes
3. Was the exposure measured in a valid and reliable way?	Yes	Yes	Yes	Unclear	Yes
4. Were objective, standard criteria used for measurement of the condition?	No	No	Yes	Unclear	Yes
5. Were confounding factors identified?	No	No	No	No	No
6. Were strategies to deal with confounding factors stated?	No	No	No	No	No
7. Were the outcomes measured in a valid and reliable way?	Yes	Yes	Yes	Unclear	Yes
8. Was appropriate statistical analysis used?	Yes	Yes	Yes	Yes	Yes
Overall appraisal:	Include (Seek further info)	Include (Seek further info)	Include (Seek further info)	Include (Seek further info)	Include (Seek further info)

**Table A3:** The Joanna Briggs Institute (JBI) Critical Appraisal Checklist for studies reporting prevalence data.

The Joanna Briggs Institute (JBI) Critical Appraisal Checklist for Studies Reporting Prevalence Data					
Author	Melsen et al. 1987 [17]	Lagana et al. 2013 [27]	Shetty et al. 2013 [28]	Kasparaviciene et al. 2014 [19]	Caruso et al. 2019 [29]
1. Was the sample frame appropriate to address the target population?	Yes	Yes	Yes	Yes	Yes
2. Were study participants sampled in an appropriate way?	Yes	Yes	Yes	Yes	Yes
3. Was the sample size adequate?	Yes	Yes	Yes	Yes	Yes
4. Were the study subjects and the setting described in detail?	Yes	Yes	Yes	Yes	Yes
5. Was the data analysis conducted with sufficient coverage of the identified sample?	Yes	Yes	Yes	Yes	Yes
6. Were valid methods used for the identification of the condition?	Yes	Yes	Yes	Yes	Yes
7. Was the condition measured in a standard, reliable way for all participants?	Yes	Yes	Yes	Yes	Yes
8. Was there appropriate statistical analysis?	Yes	Yes	Yes	Yes	Yes
9. Was the response rate adequate, and if not, was the low response rate managed appropriately?	Yes	Yes	Yes	Yes	Yes
Overall appraisal:	Include	Include	Include	Include	Include
